# Beyond kidney dialysis and transplantation: what’s on the horizon?

**DOI:** 10.1172/JCI159308

**Published:** 2022-04-01

**Authors:** Hamid Rabb, Kyungho Lee, Chirag R. Parikh

**Affiliations:** Division of Nephrology, Department of Medicine, Johns Hopkins University School of Medicine, Baltimore, Maryland, USA.

There are currently over 750,000 patients with end-stage renal disease (ESRD) in the United States. Globally, 2.6 million patients receive renal replacement therapy with either dialysis or a kidney transplant, which is estimated to double in number by 2030 (1). Kidney care was revolutionized by the invention of the dialysis machine in 1943 by Willem Kolff and the subsequent development of the arteriovenous fistula in 1960 by Belding Scribner. The first successful human kidney transplantation was performed in 1954 by Joseph Murray, teaming with John Merrill, and has since become the treatment of choice for patients with ESRD. Although there have been only incremental innovations since that time, recent exciting developments in kidney research have the potential to transform treatment beyond dialysis and transplantation. Here, we highlight five emerging approaches for ESRD.

## Wearable and bioartificial kidneys

**Figure 1:**
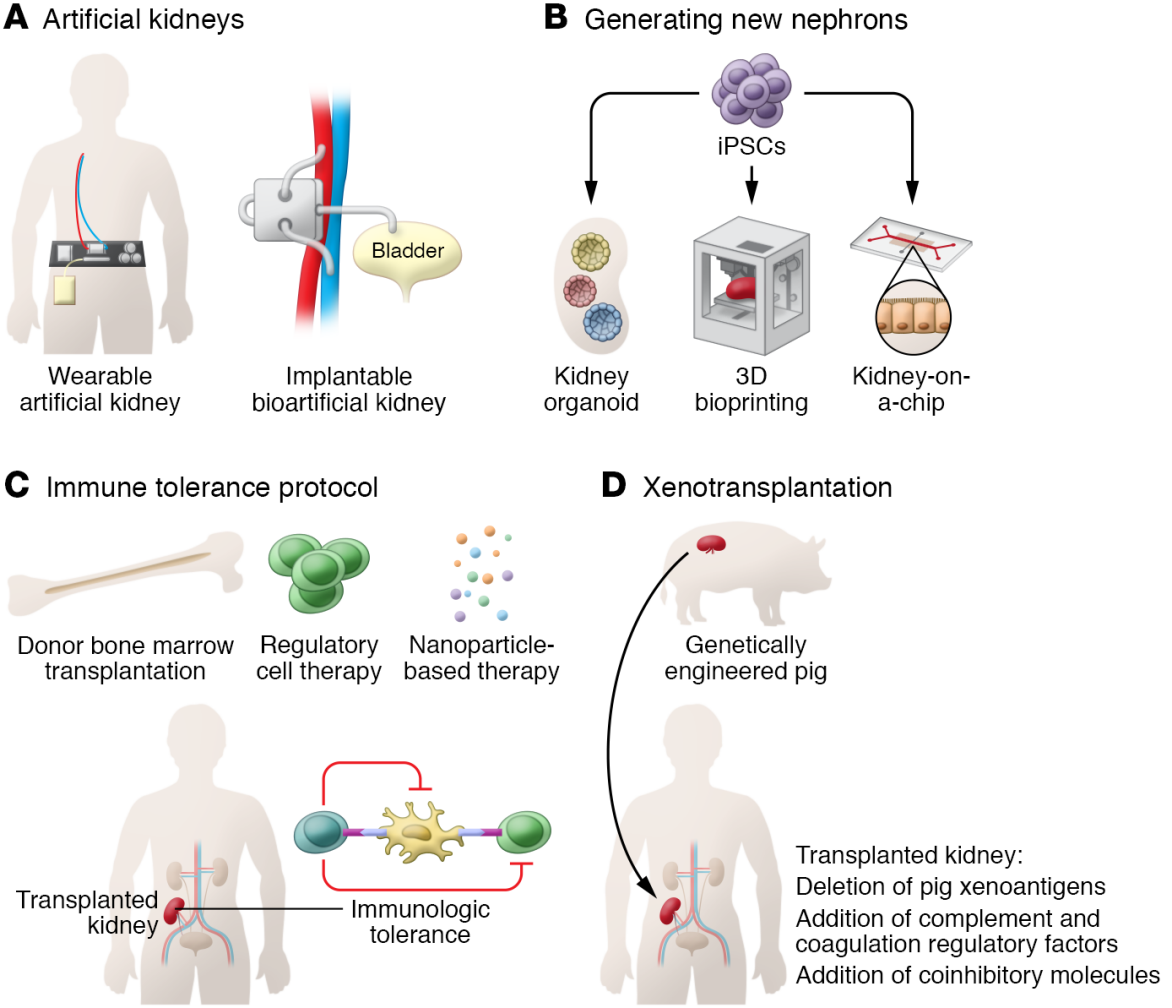
Next-generation approaches for ESRD. (**A**) The automated wearable artificial kidney is designed to perform continuous dialysis throughout the day with increased portability. The implantable bioartificial kidney is a surgically implanted device that mimics a native kidney. (**B**) The 3D cell bioprinting technique allows layer-by-layer stacking of cells and facilitates further development of both kidney organoid and kidney-on-a-chip technologies. (**C**) Clinical transplantation tolerance trials using chimerism induction approaches have made substantial progress with safety. (**D**) Pigs that have been genetically engineered, including by deletion of pig xenoantigens and insertion of protective human transgenes to induce immunologic tolerance, are currently available.

The wearable artificial kidney and implantable bioartificial kidney each have the potential to provide continuous dialysis throughout the day. They are expected to contribute to higher toxin clearance with more cardiovascular stability as well as improved quality of life. The wearable artificial kidney is a miniaturized wearable hemodialysis device with continuous dialysis capacity. The latest version is a belt-like device weighing up to 5 kg that is connected to blood vessels through catheters (Figure 1A and ref. 2). In a recent clinical trial, this device was tolerated for 24 hours without any serious complications, with effective uremic solute clearance (2). The mean ultrafiltration volume was 1 L in patients who completed assigned 24-hour dialysis, and the study participants reported higher satisfaction with the wearable artificial kidney compared with conventional hemodialysis (2). Although the clinical trial was stopped early because of technical issues, including variable flow rates and carbon dioxide bubbles in the dialysis circuit, the study provided proof of concept for the wearable artificial kidney as an alternative future dialysis option (2).

The implantable bioartificial kidney is a hybrid device that combines a mechanical blood filter made with a silicon membrane and a bioreactor containing engineered renal tubular epithelial cells. The efficient silicon membrane enables glomerulus-like filtration, and the bioreactor maintains electrolyte balance and metabolic functions. The implantable bioartificial kidney is designed to attach directly to the systemic circulation and is controlled by the patient’s blood pressure, thus eliminating the need for electrical pumps, with filtered waste removed directly into the bladder (Figure 1A). The implantable bioartificial kidney is under preclinical testing and has not yet been subjected to peer review, but the prototype silicon cartridge has been tested in canine models and showed sustainable patency without the need for an electrical pump or anticoagulants for up to one month (3). A major concern with the implantable bioartificial kidney will be the durability and clotting of the blood filter, as patients may need frequent surgery to manipulate or change the device. Moreover, the immortalized engineered tubular cells must retain stability and sustained viability in the face of the high blood shear force needed for clinical use.

## Kidney-on-a-chip

An organ-on-a-chip is an advanced microfluidics-based cell culture platform designed to mimic organ physiology. Continuous flow through a microfluidics system provides a physiological cell microenvironment, which allows long-term culturing while maintaining cell phenotypes (Figure 1B). This chip-based technique in nephrology medicine has been used to reproduce tubular structures or glomeruli. A number of different glomerulus-on-a-chip and tubule-on-a-chip models have been tested for drug screening, disease modeling, and in vivo regenerative medicine applications. Whereas traditional proximal tubule-on-a-chip models were limited by the lack of vasculature, a more advanced model with vascularization was recently created by 3D bioprinting technology. This vascularized proximal tubule model exhibited tubular reabsorption through tubular-vascular exchange, with potential applications in nephrotoxicity assessment and therapeutic development (4). An improved glomerulus-on-a-chip model that recapitulates the human glomerular filtration barrier has been developed from human podocytes and glomerular endothelial cells that are not separated by an artificial membrane, allowing direct cellular communication (5). This model reproduced selective permeability with differential clearance of albumin and inulin. Exposure to serum from patients with membranous nephropathy who have anti-podocyte autoantibodies led to the development of albuminuria in this model, whereas normal serum did not (5). Given the availability of both tubule-on-a-chip and glomerulus-on-a-chip models, future studies to develop next-generation chips combining both elements to generate a functional nephron that recapitulates both filtration and reabsorption are warranted.

## Growing a new kidney from stem cell–derived organoids

The discovery of induced pluripotent stem cells (iPSCs) has enabled the use of human PSCs and led to the development of innovative protocols for human organoid research. Since the introduction of induction protocols for human iPSCs-derived kidney precursor cells, the kidney organoid field has flourished (6–8). A recent advance in 3D bioprinting technologies further facilitated improvement in kidney organoid technologies by allowing the fabrication of tissue in an automated and spatially controlled fashion (Figure 1B). These advances improved the quality of organoids and also provided high throughput and reproducibility (9). Encouraging preliminary data suggest a potential use of kidney organoids as kidney disease models (7, 8). However, sophisticated analyses using single-cell RNA-Seq revealed that current organoids are limited to developing kidneys that do not mature beyond the second trimester stage. Thus, to model various adult human kidney diseases and for use of regenerative medicine, further research is required to generate more mature organoids (10). Thus, current kidney organoid systems do not model adult human kidneys, and further research is required to generate more mature organoids. Recent advances in understanding human kidney development through cutting-edge technologies such as single-cell RNA-Seq are expected to enable the development of more mature organoids in the future.

## Immune tolerance protocols 

## for kidney transplants

With improved immunosuppressive drugs and better HLA matching, short-term graft survival has been substantially improved over the past few decades; however, there is room for improvement in long-term graft survival. As maintenance immunosuppression can limit both graft and patient survival because of toxicity, transplantation without long-term immunosuppression has been studied (Figure 1C). The main current approach for tolerance induction is based on mixed chimerism induction through donor bone marrow transplantation or donor-derived hematopoietic stem cell transplantation. Although the number of study participants has been limited, multiple centers recently reported successful discontinuation of maintenance immunosuppression with favorable safety (11). Another promising approach is regulatory cell–based therapy, which takes advantage of their tolerogenic effect. In a recent single-arm multicenter clinical trial (The ONE Study), 40% of patients were successfully weaned to tacrolimus monotherapy over the 60-week period by use of cell-based medical products containing Tregs, DCs, or macrophages (12). The study group showed a favorable safety profile, with fewer infectious complications compared with the standard-of-care group (12). Randomized, controlled clinical trials based on this study are currently underway (ISRCTN11038572). Using nanoparticles for selective delivery of donor alloantigens or immunosuppressants to antigen-presenting cells has also been proposed as a strategy to induce immune tolerance in transplantation. Nanomaterials per se are known to not induce activation of antigen-presenting cells and can be easily customized, enabling delivery of their therapeutic cargo with minimal immunogenicity and high uptake. One such example is the administration of rapamycin-containing silicon nanoparticles to selectively target DCs in mice and nonhuman primates, which has demonstrated promising results of splenic Treg expansion and high kidney transport (13).

## Xenotransplantation

Although more than 90,000 patients in the United States are currently on the waiting list for kidney transplants, only 20,000–25,000 kidney transplantations are performed annually, with the continuous shortage of donor organs being the main barrier to improving ESRD outcomes (14). Xenotransplantation using domestic pigs has been considered a promising future strategy to alleviate the organ shortage. Since humans have preformed antibodies against porcine xenoantigens, which cause hyperacute rejection, triple-knockout pigs lacking three major xenoantigens (αGal, Neu5Gc, and SDa) were developed and are now used as a xenotransplantation model. Based on the triple-knockout pig model, additionally advanced genetically engineered pigs that have protective human transgenes, including those for costimulatory molecules and coagulation pathway and complement cascades, are currently available and expected to further improve xenotransplantation outcomes (Figure 1D). With remarkable advances in gene-editing technologies through the CRISPR/Cas9 technique, xenotransplantation outcomes in nonhuman primates have been continually improved, achieving recipient survival beyond one year (15). The first peer-reviewed, clinical-grade kidney xenotransplantation study was recently published (16). A human brain-dead decedent underwent bilateral nephrectomy followed by xenotransplantation of kidneys from pigs harboring ten genetic modifications, including human complement inhibitor genes, anticoagulant genes, and immunomodulatory genes, as well as the three xenoantigens. The recipient remained hemodynamically stable, with a viable graft, excreting urine until study termination 72 hours after xenotransplantation. However, the xenografts failed to achieve creatinine clearance and showed histologic evidence of thrombotic microangiopathy with uncertain etiology. Given a possible complement-mediated mechanism for thrombotic microangiopathy in xenografts, this outcome raises the question of whether additional genetic modifications targeting the complement pathway are required. Ethical, social, and religious concerns regarding xenotransplantation also need to be addressed in future studies. If xenotransplantation becomes clinically available, educational strategies for the general public and potential kidney transplant patient candidates and their families are likely to be required for its successful application.

Given the steadily increasing number of patients with ESRD, we have a pressing need for discoveries and therapeutic innovations. Clinical application of the technologies for ESRD will require more time, resources, and effort, however, they have the potential to make dramatic improvements in the care of patients with kidney failure.

## Version 1 04/01/2022

Electronic publication
